# Reflex Actions Determined by Constant and Continuous Electric Currents

**Published:** 1869-03-01

**Authors:** 


### *Reflex Actions Determined by Constant and Continuous Electric Currents.*

BY DR. ONIMUS.

The author considers the reflex contractions which are produced in muscles remote from those which receive the divisions of the electrified nerve.

In the cases in which the motor and the sensitive nerves are intact, it is observed that the descending or centrifugal current produces reflex contractions at the close, and the ascending or centripetal produces reflex contractions at the opening.

In the cases in which the sensitive nerves are paralyzed (by section of the posterior roots) it is observed that the ascending current produces nothing. In the cases in which the motor nerves are paralyzed (by the administration of curare, after ligature of the artery of the limb which is to be protected from the action of the poison) the same results are obtained as when the nerves are intact.

One question remains which the author proposes to solve, viz.: Is the action of the current as a cause of reflex action continuous, or does it act upon the separated parts only at the moment when the contractions appear?

